# Ordinal Logistic Regression Analysis in Determining Factors Associated with Socioeconomic Status of Household in Tepi Town, Southwest Ethiopia

**DOI:** 10.1155/2022/2415692

**Published:** 2022-02-03

**Authors:** Mesfin Esayas Lelisho, Abebe Argaw Wogi, Seid Ali Tareke

**Affiliations:** ^1^Department of Statistics, College of Natural and Computational Science, Mizan-Tepi University, Tepi, Ethiopia; ^2^Department of Statistics, College of Natural and Computational Science, Ambo University, Ambo, Oromia, Ethiopia

## Abstract

**Background:**

Socioeconomic status (SES) refers to an individual's or group's social position or class, which is often determined by a combination of education, income, and occupation. Knowing factors that affect the SES of the society might help to take action and improve their economy. In addition, using an ordinal logistic regression model for ordered SES outcomes will yield suitable results and conclusions. This study aimed to utilize an ordinal logistic regression model to find the factors associated with SES for households in Tepi town, Southwest Ethiopia.

**Methods:**

The community-based cross-sectional study was carried out in Tepi town, southwest Ethiopia, with data collected from 382 households using a simple random sample technique. The ordinal logistic regression models were evaluated and contrasted for proper accounting of ordinal form. In addition, to come up with a better model, we compared fitted ordinal logistic models with the likelihood-ratio test and AIC criteria. We performed data analysis using STATA version 16.

**Results:**

Of all 382 household heads, 170 (45.5%), 120 (31.4%), and 92 (24.1%) were at low, medium, and high SES of households, respectively. According to the result of the multivariable, partial proportional odds model (PPOM), age, education level, family size, and the saving habit were significantly associated with the SES of households at a 5% level of significance.

**Conclusions:**

According to the findings of this study, ordinal regression may be a better option in the event of the ordinal form of the outcome. Furthermore, PPOM may be a preferable option if any of the covariates violate the proportionality requirement. Based on the result of this study, the most likely associated indicators with the SES of families in Tepi town, southwest Ethiopia, were family size, age, saving habit, and education level. It is recommended that action should be taken to improve the SES of households.

## 1. Introduction

Socioeconomic status (SES) is a composite assessment of a person's economic and sociocultural circumstances. It is a complicated evaluation based on a range of methods that considers a person's work experience and their economic and social position in relation to others, as assessed by income, education, and employment [1–3]. When determining a family's SES, the household income, education, and occupation of the earners are considered [4–7].

The modified Kuppuswamy scale [8, 9], which is often used to determine SES in urban and rural contexts, is made up of a composite score that includes the family head's education, occupation, and the family's monthly income and gives a score between 3 and 29 [10–12]. Modified Kuppuswamy socioeconomic scale updated for the year 2019 [8] was the most commonly used scale for determining the SES of an urban family. Some researchers believe that wealth is the most powerful and reliable predictor of health [13–16], because it mediates the impact of other SES variables to some extent [17].

In developing countries like Ethiopia, demographic factors such as family size, sociocultural circumstances, religion, level of education, age, marital status, gender, and occupation are the most popular factors that affect the economic activities of society [18]. Previous research from Colombia [19] found that socioeconomic factors such as education level and agricultural income play a role in the adoption of sustainable practices in smallholder households. Income, education, employment, community safety, and social support are all social and economic factors that can have a significant impact on how well and how long we live. These factors influence our ability to make healthy choices, afford medical care and housing, manage stress, and many other things [20].

Application of binary logistic regression (BLR) for a variable with natural order might lead to misleading results and interpretation [21]. In addition, for situations with a polychotomous outcome variable, the two possible categories are multinomial and ordinal [22–26]. The multinomial logistic regression model cannot be used if the dependent variable is categorized according to its order of magnitude. There are several ordinal logistic regression models such as the proportional odds model (POM), two versions of the partial proportional odds model-without restrictions (PPOM-UR) and partial proportional odds model-with restrictions (PPOM-R), continuous ratio model (CRM), and stereotype model (SM) [27]. Among various OLR models, the most frequently used OLR model in practice is the constrained cumulative logit model called the proportional odds model [22, 28].

For assessing ordinal response variables, the proportional odds model (POM) is the most widely used logistic regression model [27, 29]. In several previous studies, the OLR model is frequently used when the response variable is ordinal [30–32]. Ordinal models are more effective at providing generalizing visualizations that compare the impact of independent variables at the class level. The identification of factors associated with SES was the interest of this study with the ordered category. Hence, we applied ordinal logistic regression (OLR) by assuming ordered categories of SES as low, medium, and high.

Despite the fact that numerous research studies on the use of ordinal logistic regression have been performed throughout the world, there is no study that considers the ordered outcome of SES. Furthermore, researching family SES is a critical topic since it is closely connected to effects on a young child's cognitive, language, social, physical, and emotional development, among other things, but has not been considered. The main goal of this study was to identify the predictors of SES for households in Tepi town, Southwest Ethiopia, by developing an ordinal logistic regression model. This will help the population to take into consideration the factors that affect the SES of the society and to take action to improve their economy.

## 2. Methods

### 2.1. Study Design and Setting

The cross-sectional study was conducted at Tepi town, Southwest Ethiopia. In the current investigation, the primary data were collected from the sample households by using questionnaires and interviews. The self-administered questionnaire was developed, and data from 382 households were obtained by using a simple random sampling technique.

### 2.2. Sample Size Determination

One of the first things to think about when planning a sample survey is the sample size. With ordinal logistic regression, the general formula for sample size determination, which is developed by Walters [33], is as given as follows:

(1) $$\begin{array}{c} n=\frac{6{\left(Z_{\alpha /2}+Z_{\beta }\right)}^{2}}{\log\;{\left(OR\right)}^{2}\left[1-\sum_{i=1}^{k}{\bar{\pi }}_{i}^{3}\right]}, \end{array}$$

where ${\bar{\pi }}_{i}$ is the average of probabilities for the *i*^*th*^ category. At the significance level of 95%, *α* = 0.05 and *Z*_*α*/2_ = 1.96. Using a pilot survey from 20 participants, and based on the gender of HOF, we found that the regression coefficient is *β* = 0.0745. Furthermore, by assuming the power *β*=20%=0.20, then *Z*_*β*_=*Z*_0.8_=0.84, and 1 − *β*=0.8; then, we have

(2) $$\begin{array}{c} \log\;{\left(\text{OR}\right)}^{2}=\log\;{\left(e^{\beta }\right)}^{2}=0.2326\text{ and }\sum_{i=1}^{5}{\bar{\pi }}_{i}^{3}={\left(0.50\right)}^{3}+{\left(0.35\right)}^{3}+{\left(0.15\right)}^{3}=0.1302, \end{array}$$

where ${\bar{\pi }}_{i}=\pi_{iM}+\pi_{iF}/2$, which is the average probability for men and women.

(3) $$\begin{array}{c} \log\;{\left(\text{OR}\right)}^{2}=\log\;{\left(e^{\beta }\right)}^{2}=0.150\text{ and }\sum_{i=1}^{3}{\bar{\pi }}_{i}^{3}={\left(0.50\right)}^{3}+{\left(0.35\right)}^{3}+{\left(0.15\right)}^{3}=0.1713. \end{array}$$

Finally, the required sample size would be obtained as follows:

(4) $$\begin{array}{c} n=\frac{6{\left(1.96+0.84\right)}^{2}}{0.150\left(1-\sum_{i=1}^{3}{\bar{\pi }}_{i}^{3}\right)}=\frac{47.04}{0.150*\left(1-0.1713\right)}=\frac{47.04}{0.1234}=381.198\approx 382. \end{array}$$

### 2.3. Study Variables, Data Collection Tools, and Measurements

The response variable for this study was SES, which is categorized as follows:

(5) $$\begin{array}{c} Yi=\left\{\begin{array}{cc} 0, & \text{low,}\\ 1, & \text{Medium,}\\ 2, & \text{High.} \end{array}\right. \end{array}$$

To assess SES, we used the modified Kuppuswamy socioeconomic scale updated for the year 2019 [12]. This is the most often used scale for determining an urban family's SES. The parameters were modified based on the education and occupation status of the HOF and the disposable income of the whole family, total from all the sources. According to the Kuppuswamy socioeconomic scale updated for 2019, the scores of the three parameters are as follows: education of household head (score: 1 = Illitrate, 2 = Primary school, 3 = Middle school, 4 = High school, 5 = Intermidiate/Diploma, 6 = Graduate, 7 = Proffessional degree); the occupation of household head (score: 1 = Unemployed, 2 = Unskilled worker, 3 = Semiskilled worker, 4 = Skilled worker, 5 = Clerical/Shop/Farm, 6 = Semi professional, and 10 = Professional); finally, the monthly income of the family (overall family income) (score: 1 = ≤2,640,2 = 2,641-7,886, 3 = 7,887-13,160, 4 = 13,161-19,758, 6 = 19,759-26,354, 10 = 26,355-52,733, and 12 = ≥52,734). The total score ranges from 3 to 29. Then, this score can be classified into five classes of socioeconomic class as follows: upper (26–29), upper-middle (16–25), lower-middle (11–15), upper-lower (5–10), and lower (<5). Based on this classification, we had recategorized the SES into the low, medium, and high classes as follows: high SES (upper class), medium SES (upper-middle and lower-middle classes), and low SES (upper-lower and lower classes). Thus, SES is an ordinal response variable grouped from a continuous variable.

Explanatory variables for this study were as follows: sex of HOF (male and female), age (below 30, 30–45, and above 45), marital status (single, married, widowed, and divorced), family size (≤2, 3–4, and ≥5), saving habit (no and yes), saving methods (traditional and modern), level of education (no formal education, primary, secondary, diploma, and higher), and religion (orthodox, Muslim, protestant, and others).

### 2.4. Method of Statistical Data Analysis

Frequency and percentages were used to highlight descriptive results. The chi-square test/Fisher's exact test was used to analyze the relationship between qualitative independent variables and response variables. Furthermore, to identify the factors associated with an ordinal form of SES, variables determined to be significant at a 25% [34, 35] level in crude association analysis (univariable analysis) were used as a subset of covariate stepwise ordinal logistic regression. For the proportionality assumption, the Brant test was applied [36]. The Hosmer test for goodness of fit was used to evaluate the model's performance [37].

#### 2.4.1. Statistical Models

To account for the ordinal nature of outcomes, various ordinal logistic regression models exist. The logits of these various ordinal regression models are formed in a variety of ways, for instance, POM (cumulated higher categories compared with remaining cumulated lower categories), CRM (cumulated higher categories compared to lower category only), and ACM (between any of two consecutive categories). As a result, each form of the logit has advantages and disadvantages; one can utilize the models based on their requirements. The proportional odds model (POM) is frequently utilized in epidemiological and biological applications. However, the continuation ratio model is also utilized on occasion [14, 38]. Our research objective of the statistical inquiry is centered on the decision of POM and CRM models. As is clear, the interpretation we do under POM would be more rational and understandable in the case of SES. If the condition of proportionality is breached, the model of PPOM could be a preferable option [39, 40]. Furthermore, the likelihood ratio test and AIC were used to evaluate the choice between POM and PPOM.

#### 2.4.2. Proportional Odds Model (POM)

Assumption of POM assures that the odds ratios are identical for all categories. The POM is utilized if the log odds ratio across the cut points is the same, i.e., the proportional odds assumption is met. It is the most widely used model, which was first introduced as a cumulative logit model by Walker and Duncan [41] but later renamed as proportional odds model by McCullagh [14]. As previously stated, each household's SES (Y) observation is classified into one of three groups. Similarly, covariates (*xi*) refer to the vector of covariates with dimension *p* (*i*= 1,  2....*p*), which contains the observation on all *p* independent variables. As a result, we may express the dependency of the response variable (*Y*) on explanatory variables *xi* as follows:

(6) $$\begin{array}{c} P\left(Y\geq y_{j}|x\right)=\frac{1}{1+\exp\left(-\alpha_{j}-x_{i}^{\prime }\beta \right)},\;j=1,2,3. \end{array}$$

On the other hand, it can also be described as follows:

(7) $$\begin{array}{c} \log\left[\frac{P\left(Y\geq y_{j}|x\right)}{1-P\left(Y\geq y_{j}|x\right)}\right]=\alpha +x_{i}^{\prime }\beta ,\text{ for},\;j=1,2,3, \end{array}$$

where *P*(*Y* ≥ *y*_*j*_) is denoted as the cumulative probability of an event (*Y* ≥ *y*_*j*_);  *αj*  is the respective constant term/intercepts; and *β* is the vector of regression coefficients with the dimension of (*p* by 1) that corresponds to the *x*_*i*_ covariates.

#### 2.4.3. Partial Proportional Odds Model (PPOM)

A PPOM may be employed if the assumption of identical log odds ratio under POM is not met for the factors [42]. The unconstrained PPOM was chosen over the constrained PPOM due to a lack of prior knowledge or opinions about limits and the availability of computer resources [43]. The PPOM permits nonproportional odds for a subset of q of the p-predictors (q ≤ p). We may define the unconstrained PPOM cumulative probability as follows:

(8) $$\begin{array}{c} P\left(Y\geq y_{j}|x\right)=\frac{1}{1+\exp\left(-\alpha_{j}-x_{i}^{\prime }\beta +t^{\prime }\gamma_{j}\right)},\;j=1,2,3, \end{array}$$

where *xi*  is denoted as a vector of (*p* by 1) that contains the values of observation *i* on the entirely *p* independent variables, and *β* is a vector of regression coefficients with dimension (*p* by 1) associated with *p* variables. Moreover, *t*′ is a vector of *q* covariates (1 by *q*) that contains the values of observation *i*  on that subset of the *p* independent variables for which assumption of proportionality is either not met or is to be tested, and *γ*_*i*_ is the vector of regression coefficients with dimension (*q* by 1), which is associated with the *q* covariates. As a result, *t*′*γ*_*j*_ is the increase, which is associated with  *jth* cumulative logit (1  ≤  *j* ≤ 3), where *γ*_1_ = 0. If values of *γ*_*i*_=0 for all *j*, then this model reduces to POM.

## 3. Results

This study was carried out to identify determinants of the SES of households through analyzing the socioeconomic and demographic factors. In this study, both descriptive and inferential analyses have been investigated to identify the determinants of the SES of households in Tepi town, Southwest Ethiopia. Accordingly, the study used 382 households.

### 3.1. Descriptive Statistics

Out of the 382 households in this study, 279 (73.0%) were men and 103 (27.0%) were women. Regarding the age of participants, a large percentage, about 151 (39.5%) of study subjects, were in the age-group between 25 and 45 years followed by 141 (37%) of participants who were below 25 years. Out of the total, 75 (19.5%) of the respondents have no formal education of which 63 (84%) were in low SES. However, secondary and higher education were 115 (30%) and 66 (17.5%) of which 51 (44.4%) and 33 (50.0%), respectively, were in high SES. Regarding the religion of households, 149 (39%), 101 (26.4%), 86 (22.5%), and 46 (12.1%) were orthodox, protestant, Muslim, and other religions, respectively. When we come to saving habits of household's nearly equal proportion of households in both categories, 195 (51%) of households have no saving habit and 187 (49%) of them have a saving habit. Regarding the family size, 134 (35%) were less than two, 141 (37%) were 3–4, and 107 (28%) were more than five family members. Out of the total study households, more than half, 252 (65.9%), were married, whereas only 76 (19.9%) were single (Table 1).

**Figure 1:**
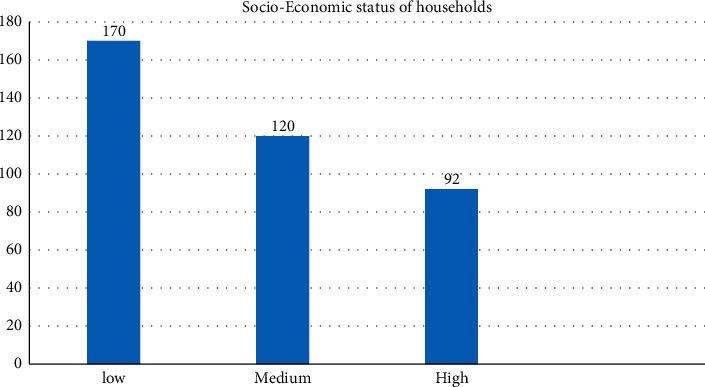
The level of the SES of households in Tepi town.

Socioeconomic status is typically broken into three levels (high, middle, and low) to describe the three places a family may fall into. In this study, we have placed a family into one of these categories, first based on the Kuppuswamy SES classification (five classes of SES scale) by assessing all of the three parameters (income, education, and occupation) and recategorized into three classes. Accordingly, of all 382 household heads, 170 (45.5%), 120 (31.4%), and 92 (24.1%) were at low, medium, and high SES of households, respectively (Figure 1).

### 3.2. Inferential Statistic Results

From the outputs in chi-square analysis (Table 1), we observed that the covariate gender, age, saving habit, education level, family size, religion, and marital status of the household head showed a significant association.

### 3.3. Univariable Analysis

In the univariable analysis, the covariates of gender, age of HOF, saving habit, education status of HOF, and family size were found to be statistically significant at the univariable level. This indicates that they are important factors that might affect the SES of the household. However, religion and marital status were not significant factors for the SES of households at a 25% level of significance. Therefore, based on this result, it is better to ignore the religion and marital status covariate and shall do our multivariable ordinal logistic analysis using the remaining factors. Hence, the effects of the covariates of gender, age of HOF, saving habit, education status of HOF, and family size on the SES of households shall better be interpreted using the multivariable ordinal logistic regression analysis.

### 3.4. Multivariable Analysis and Model Comparison

Five variables were chosen for the stepwise regression from seven available variables based on their crude association at a 25% level of significance. Before developing the multivariable ordinal logistic regression model, we have checked the collinearity and the first-order effect modifier was evaluated. However, in the current dataset, they were not present.

Except for covariate's saving habit, the proportional odds assumption was determined to be satisfactory in multivariable regression analysis for each of the investigated factors used to develop the final model. The key assumption in ordinal regression is that the effects of any explanatory variables are consistent or proportional across different thresholds, which are commonly referred to as the proportional odds assumption (parallel line test). The proportionality assumption holds if the *p* value for the parallel line test had a large *p* value. In this study, the overall proportionality assumption in this study was not violated, i.e., *p* value = 0.168. This result suggests that the proportionality assumption holds because the *p* value is large (>0.05), which is statistically insignificant. As a result, both models (POM and PPOM) were created and evaluated (Table 2).

#### 3.4.1. Result of Proportional Odds Model (POM)

The covariates of age, saving habit, education level, and family size were significant at a 5% level of significance using the multivariable POM, indicating that this was the important deterministic factor for household SES. The gender of the household head, on the other hand, had no significant effect.

At a 5% level of significance, the score test for the proportional odds assumption is insignificant, indicating that the data meet the proportional odds assumption. Single score tests of the proportional odds assumption for each covariate were performed to corroborate the conclusion about the POM assumption. The single score tests' *p* values are provided in the last column of (Table 2). The test results reveal that all the variables except the age of the household head (*p* value = 0.003) were found insignificant, i.e., satisfy the proportional odds assumption. To check further, we use PPOM to assess the data again, without concluding.

#### 3.4.2. Result of Partial Proportional Odds Model (PPOM)

Age (in years), education, family size, and saving habit were revealed to be substantially linked factors in multivariable PPOM, just as they were in POM (Table 3). The GOLOGIT2, which is the default of STATA, produces results that are similar to a series of BLR and can be similarly interpreted. The fundamental issue with both techniques' outcomes is that they incorporate far more parameters than POM. These approaches remove the parallel line requirement from all variables, even if only one or a few of them break the assumption. As a result, the study employed the AUTOFIT option with GOLOGIT2 to fit the PPOM. By doing so, the parallel line constraint relaxed only for those variables where the assumption was not justified, and the parallel line constraint was considered for the rest of the variables that satisfied the assumption [44].

Moreover, to come up with a model that best describes the dataset, we applied AIC and likelihood-ratio test. The evaluation of both models (Tables 2 and 3) demonstrated that PPOM is the preferred model, which is determined by LR and AIC (Table 4). Furthermore, the likelihood-ratio test supports this.

The result from multivariable ordinal logistic regression (Table 2) showed that the saving habit of households was statistically significant at a 5% level of significance. The estimated odds ratio (OR = 5.74, 95% CI, 2.12–15.56) indicated that those who have saving habits were 5.74 times more likely to be in high SES as compared to households having no saving practice holding all other variables constant. This suggested that saving is crucial to improve the economic level, as a result, the SES of households.

The result of the study also showed that age was significantly related to the SES of the household (OR = 3.49, 95% CI, 1.05–12.07). Household head aged 25–45 years was 3.49 times more likely to be in high SES as compared to those households aged below 25 years. In other words, the households aged between 25 and 45 years had a 3.49 times higher chance to be involved in higher SES. Education status was another significant factor that influences the SES of households in Tepi town. Households those who have education status diploma and higher were 7.862 times more likely to be in high SES as compared to those who have no formal education. Furthermore, those who have secondary education were 4.14 times more likely to be in high SES as compared to households' who have no formal education, holding other effects of other covariate's constant. Family size among households appears to be an important indicator of economic effect. The estimated odds ratio (OR = 0.76, 95% CI: 0.01–0.96) suggested that the ordered odds of subjects who have a family size of ≥5 children were 0.76 times less likely to be in high SES as compared to those who have family sizes of less than two, keeping all other covariates fixed.

#### 3.4.3. Evaluation of the Fitted Model

The goodness-of-fit test shows that deviance statistics with (*p* value = 1.000) is large. This indicated that the model fits data well. Furthermore, Nagelkerke's *R* = 0.647 suggested that 64.7% of the variations among response variables were explained by existing explanatory variables in the model, and the remaining 35.3% were accounted for by error terms and unseen factors (Table 2).

## 4. Discussion

The purpose of this study was to use an ordinal logistic regression model to identify factors associated with a household's socioeconomic status (SES) under the assumption of ordered categories. This study attempted to implement Kuppuswamy SES classification (five classes of SES scale) by assessing all three parameters (income, education, and occupation) to classify households' SES. According to the Kuppuswamy scale (KWS) of socioeconomic classes, the parameters such as the education, occupation of HOF's, and the total family income from all sources were modified [12]. Accordingly, households were with KWS less than 10 (low), KWS between 10 and 25 (medium), and KWS greater than or equal to26 (high). Our findings revealed that out of the 382 household heads who took part in the study, 170 (45.5%), 120 (31.4%), and 92 (24.1%) had low, medium, and high SES, respectively. This suggested that nearly half of the participants fell into a lower socioeconomic class. Furthermore, our study also revealed that based on the final selected model, PPOM, age, education level, family size, and saving habits were statistically significant determinants of SES.

Previous studies [45] reported the age of respondents as an important factor linked with SES. In line with this report, this study showed that middle age was substantially linked to the improvement of household SES. In a previous study, it was found that the primary breadwinner's age has a significant positive impact on multidimensional energy poverty and that increasing age exacerbates energy vulnerability [19]. This is also supported by our study, which found that older age-groups were less likely to have higher SES than younger age-groups.

Moreover, education status was also reported as a key factor of SES. Those who have an education level of diploma and higher were more likely to be in higher socioeconomic class. Similarly, the prior study reported that individuals with low SES are typically those with low educational accomplishments and/or low household income [46]. Previous research has found that education is a significant income determinant and, as a result, a growth factor, regardless of whether education can increase productivity [47].

Family size was a statistically significant predictor of household socioeconomic status. Previous studies reported that family size is an important factor in determining multidimensional energy poverty [19]. According to their study as family size increases, multidimensional energy poverty decreases. As a result, larger families are more vulnerable to energy poverty than smaller families. In contrast to their findings, this study found that as family size increases, the likelihood of having a high SES decreases. This could be attributed to the fact that a large family size leads to more debts, consumption, and thus a lower SES.

Developing countries have low income and savings rates, trapping them in poverty traps and perpetuating the vicious cycle of poverty [48]. Saving habit has a great role to improve one's income so does the SES. Another study also revealed that saving has a substantial effect on the improvement of the economic condition [49]. According to Loibl et al., the habit of saving plays a significant role in daily financial activity decisions [50]. Saving in a consistent and good manner is critical to a household's financial independence and economic stability. As a result, one's income rises, as does one's social status. In line with these reports, the current study revealed that saving habit has a significant effect on the SES of households, and those who have a habit of saving had higher odds of being at higher SES. According to previous studies from Colombia, [51] reported that socioeconomic factors, education level, income from agriculture, access to credit, and level of cooperative membership play a determinant role in the adoption of sustainable practices in smallholder households. This could be due to the fact that those with higher education and income have more decision-making power in their business activities.

In SES, ordinal categories such as low, medium, and high are the result of the grouping of quantitative data. Dichotomization or discarding the order, like changes in origin and scale, has disadvantages [52]. POM appeared to be adequate in our investigation, as the total model did not significantly violate the proportional odds assumption. One of the factors, however, was discovered to violate this premise. According to a comparable study from India, when POM and PPOM were compared, PPOM was shown to be more appropriate for some factors that violated the score test [53]. However, in the current investigation, the *p* value of the overall model's score test was extremely low, necessitating the use of a single score test for each covariate. These tests reveal that just the household head's gender contradicts a key POM assumption, potentially resulting in inaccurate results. There are no clear criteria for when the proportional odds assumption should be modified either on theoretical considerations or on empirical tests [27]. In this study, the AIC and likelihood-ratio test supported the PPOM during the investigation of this possibility, whether to employ POM or PPOM. GOLOGIT2 availability with AUTOFIT syntax in STATA makes choosing the right model between POM and PPOM much simpler [39, 43].

## 5. Conclusions

This study looked at identifying factors associated with socioeconomic status (SES) by applying ordinal logistic regression. According to the findings of this study, ordinal regression may be a better alternative in the case of the ordinal form of the outcome. Furthermore, PPOM may be a preferable option if any of the covariates violate the proportionality requirement. This almost certainly ensures that the result and the inferences and implications that follow are correct. Finally, the most likely associated indicators with the SES of families in Tepi town, Southwest Ethiopia, were family size, age, saving habit, and education level. This suggests that the application of the OLR model for ordered outcomes is a preferable option, and in addition, improvement of SES based on significant covariates is needed.

## 6. Limitation

This study attempted to assess factors associated with household SES in Tepi town, Southwest Ethiopia, using ordinal logistic regression models such as POM and PPOM. This is one of the study's strengths. The study, on the other hand, has some limitations. The data were gathered through a self-administered question, which may or may not address all of the town's issues. In addition, some variables are not included in this study, such as cultural factors, that might have the power of determining SES. Furthermore, the researcher recommends that future work should consider other types of ordinal logistic regression.

## Figures and Tables

**Table 1:** Frequency distribution table of the respondents' profile.

Variables	Categories	Total *N* (%)	SES of households			^1^ *p* value
			Low	Medium	High	
Gender	Male	279 (73)	164 (58.8)	72 (25.8)	43 (15.4)	0.003
	Female	103 (27)	57 (55.6)	37 (35.6)	9 (8.8)	

Age	Below 25	141 (37)	99 (70.2)	38 (26.9)	4 (2.9)	0.002
	25–45	151 (39.5)	64 (42.4)	53 (34.8)	34 (22.8)	
	Above 45	90 (23.5)	63 (69.2)	19 (20.5)	9 (10.3)	

Saving habit	No	195 (51)	96 (49.2)	57 (29.3)	42 (21.5)	<0.001
	Yes	187 (49)	108 (57.8)	66 (35.3)	13 (6.9)	

Education status	No education	75 (19.5)	63 (84)	10 (1.3)	2 (2.7)	<0.001
	Primary	126 (33)	100 (79.4)	23 (18.3)	4 (3.2)	
	Secondary	115 (30)	23 (20.0)	41 (35.6)	51 (44.4)	
	Diploma and higher	66 (17.5)	13 (19.7)	20 (17.4)	33 (50.0)	

Family size	≤2	134 (35)	31 (23.0)	35 (26.1)	68 (50.7)	<0.001
	3–4	141 (37)	30 (21.3)	35 (24.8)	76 (53.8)	
	≥5	107 (28)	57 (53.3)	35 (32.7)	15 (14.0)	

Marital status	Single	76 (19.9)	38 (50.0)	33 (43.4)	5 (6.6)	0.039
	Married	252 (65.9)	95 (37.7)	42 (16.7)	110 (43.6)	
	Widowed	31 (8.2)	19 (61.3)	10 (32.3)	2 (6.4)	
	Divorced	23 (6)	16 (69.6)	5 (21.7)	2 (8.7)	

Religion	Orthodox	149 (39)	101 (67.8)	36 (24.2)	12 (8.0)	0.042
	Protestant	101 (26.4)	36 (35.6)	49 (46.5)	7 (6.9)	
	Muslim	86 (22.5)	49 (56.9)	26 (30.2)	11 (12.7)	
	Others	46 (12.1)	41 (89.1)	3 (6.5)	2 (4.4)	

Total		382	170 (44.5)	120 (31.4)	92 (24.1)	

^1^ *p*value was calculated by the chi-square test.

**Table 2:** Parameter estimates of POM using SES status as response with three-ordered categories.

Variables	Categories	Estimate	S. E	*p* value	OR	95% CI for OR		Score test
Gender	Male				1			0.003
	Female	0.357	0.481	0.326	1.43	0.56	3.67	

Age	Below 25							0.225
	25–45	1.250	0.626	0.004	3.49	1.02	11.91	
	Above 45	−0.457	0.573	0.424	0.63	0.21	1.95	

Saving habit	No				1			0.523
	Yes	1.747	0.509	<0.001	5.74	2.12	15.56	

Education status	No formal education				1			0.336
	Primary	0.510	0.517	0.325	1.67	0.60	4.59	
	Secondary	1.421	0.658	0.016	4.14	1.14	15.04	
	Diploma and higher	2.062	0.853	<0.001	7.86	1.48	41.84	

Family size	≤2				1			0.821
	3–4	−1.246	1.142	0.069	0.29	0.03	2.70	
	≥5	−2.126	1.167	<0.001	0.12	0.01	1.18	

Parallel line test: 0.068, goodness-of-fit test of overall model: deviance, *p* value = 1.00, Nagelkerke's *R* = 0.647, S.E: standard error, AOR: adjusted odds ratio, CI: 95% confidence interval for coefficients.

**Table 3:** Parameter estimates of PPOM using SES as response with three-ordered categories.

Variables	Categories	Estimate	S. E	*p* value	OR	95% CI for OR	
Gender	Male				1		
	Female	0.367	0.481	0.326	1.43	0.57	3.63

Age	Below 25				1		
	25–45	1.270	0.626	0.004	3.49	1.05	12.07
	Above 45	−0.357	0.573	0.424	0.66	0.23	2.11

Saving habit	No				1		
	Yes	1.747	0.509	<0.001	5.74	2.12	15.56

Education status	No formal education				1		
	Primary	0.540	0.517	0.325	1.67	0.62	4.74
	Secondary	1.421	0.658	0.016	4.14	1.14	15.04
	Diploma and higher	2.072	0.853	<0.001	7.86	1.55	40.64

Family size	≤2				1		
	3–4	−1.246	1.142	0.074	0.46	0.03	2.70
	≥5	−2.326	1.167	<0.001	0.76	0.01	0.96

Threshold	SES 1	−4.464	1.365	0.001	0.012	0.001	0.167
	SES 2	−1.322	1.346	0.326	0.267	0.019	3.729

OR: adjusted odds ratio.

**Table 4:** A comparison of the developed ordinal logistic regression models.

Fitted model	LR	AIC
POM	−533.11	1432.28
PPOM	−528.16	1423.55

## Data Availability

The datasets used in this study are available from the corresponding author on reasonable request.

## Abbreviations

AIC: Akaike information criteria
BLR: Binary logistic regression
HOF: Head of the family
KWS: Kuppuswamy scale
LR: Likelihood ratio
OR: Odds ratio
POM: Proportional odds model
PPOM: Partial proportional odds model
SES: Socioeconomic status
SNNPR: Southern Nations, Nationalities, and People's Region.
